# A rare association of rheumatoid arthritis and primary biliary cirrhosis treated with rituximab: a case report

**DOI:** 10.1186/1752-1947-7-99

**Published:** 2013-04-09

**Authors:** Faiza Lazrak, Fatima Ezzahra Abourazzak, Khadija Berrada, Nadira Kadi, Samia Manssouri, Taoufik Harzy

**Affiliations:** 1 Department of Rheumatology, University Hospital Hassan II, Fez, Morocco; 2 University Sidi Mohammed Ben Abdellah, Fez, Morocco

## Abstract

**Introduction:**

Primary biliary cirrhosis is an autoimmune disease that tends to progress to fibrosis and cirrhosis with hepatic failure. Primary biliary cirrhosis is often associated with other non- hepatic autoimmune diseases. An association with rheumatoid arthritis has been suggested to coexist in 1.8% to 5.6% of patients with primary biliary cirrhosis, but data supporting this association are scarce. The etiologic and pathogenetic mechanisms are not yet fully understood and several factors have been implicated. The therapeutic management must consider the two pathologies.

**Case presentation:**

We describe the case of a 60-year-old Moroccan woman with severe erosive rheumatoid arthritis and primary biliary cirrhosis treated with rituximab. During treatment, we observed a good clinical and biological response of her rheumatoid arthritis but persistent abnormal liver function tests.

**Conclusion:**

B cells seem to play a major role in the pathogenesis of both rheumatoid arthritis and primary biliary cirrhosis. Additional studies are necessary to better determine the therapeutic role of rituximab in both diseases.

## Introduction

Primary biliary cirrhosis (PBC) is an autoimmune disease characterized by chronic destruction of bile ducts that can lead to liver cirrhosis. It can be accompanied or preceded by various autoimmune disorders [1]. PBC can be associated with Sjögren’s syndrome in 70% of patients [2], autoimmune thyroid disease in about 10%, and systemic sclerosis in 15% [3]. The association of PBC with rheumatoid arthritis (RA) is exceptional, and the true prevalence of PBC in RA is not well known [4]. This may impose several therapeutic and diagnostic challenges. In both diseases, it was observed that B cells played a prominent role in pathogenesis [5]. We describe an unusual case of a patient with PBC and RA treated with rituximab.

## Case presentation

A 60-year-old woman was diagnosed with PBC in 2004. The diagnosis was based on the pathology of a liver biopsy that revealed signs of nonsuppurative cholangitis without fibrosis or cirrhosis and on laboratory tests showing high levels of alkaline phosphatase (four times the upper normal limit) and gamma-glutamyltransferase (three times the upper normal limit); positive type 2 anti-mitochondrial antibodies (1:640) serological tests were negative for hepatitis B and C. She was treated with ursodeoxycholic acid (UDCA) at a dose of 12mg/kg (600mg/day), with partial regression of her hepatic disorder. One year later, our patient presented with a deforming polyarthritis of her small and large joints. A physical examination on admission found synovitis in nine joints, seven painful joints, bilateral ulnar deviation hand deformities, Z-thumb deformity, and a limitation of motion in her wrists, shoulders and left hip.

Laboratory tests showed that our patient had an inflammatory syndrome with an erythrocyte sedimentation rate of 81mm at the first hour and a C-reactive protein level of 14mg/L. Her rheumatoid factor was positive at 143IU/L, and she had an anti-citrullinated protein antibody level of 798UI/mL. Radiographs showed erosions of her metacarpophalangeal and metatarsophalangeal joints, bilateral carpus and tarsus, atlantoaxial dislocation, and left coxitis. Based on this clinical, biological and radiological evidence, a diagnosis of active and severe RA was made. Our patient was treated with a low dose of methotrexate (MTX; 7.5mg/week) associated with rituximab (two doses of 1000mg separated by two weeks), which demonstrated good efficiency in her arthritis after five months of follow-up (Disease Activity score-28 of 2.8), but her abnormal liver function tests persisted.

## Discussion

The association of PBC with RA is rare and not clear, although several studies since the 1970s have pointed to a possible association. However, the true prevalence of PBC in RA is not well known. A study of 172 patients with PBC confirmed that a relatively large proportion of patients with PBC have an autoimmune rheumatic disease [6]. We found that scleroderma was the most common connective tissue disease associated with PBC [3-6]. In a Japanese review, three of the 54 patients with PBC had polyarthritis, although the authors did not specify whether or not RA criteria were fulfilled [7]. The studies suggest that the prevalence of RA in PBC patients is between 1.8% and 5.6% [6]. Further studies are needed to determine the relationship between these two diseases; in particular, an investigation of PBC occurrence in large RA patient cohorts is needed.

The case described here illustrates the possible association of RA and PBC. The etiologic and pathogenetic mechanisms of PBC are not yet fully understood and several factors have been implicated. Among them, it has been suggested that B cells may have different roles in the induction of PBC [3-8]. Both B-cell and T-cell responses to the E2 subunit of the inner mitochondrial membrane enzyme pyruvate dehydrogenase complex have been documented in PBC and implicated in its pathogenesis [9]. The serological hallmark of PBC is the presence of anti-mitochondrial autoantibodies, especially to the E2 subunit of pyruvate dehydrogenase complex, which are present in 90% to 95% of patients with PBC. In addition, patients with PBC often have elevated serum levels of total immunoglobulin M, and B cells from patients with PBC produce significantly greater amounts of immunoglobulin M when stimulated with CpG-B compared with those from healthy controls [10]. Furthermore, treatment with anti-cluster of differentiation 20 monoclonal antibody resulted in amelioration of liver inflammation, supporting a potential mechanism of action for B-cell-targeted therapies in PBC.

The association between PBC and RA has raised questions about the hepatotoxicity of RA drugs [11]. In this case, we examined the effects on safety and immunological selective B-cell depletion using rituximab (an anti-cluster of differentiation 20 monoclonal antibody) with low-dose MTX in a patient with PBC and RA.

MTX has been previously tested as a treatment of PBC, especially when conventional UDCA monotherapy is ineffective [4-11]. Conflicting data have been reported, but a recent 10-year follow-up study suggested that MTX plus UDCA was more effective than UDCA alone in terms of survival without liver transplantation [4].

In an open study, six patients with PBC and incomplete responses to UDCA were treated with two doses of 1000mg rituximab two weeks apart and followed for 52 weeks. The results of this study showed that rituximab was as safe and well-tolerated in patients with PBC as in patients with other indications, suggesting that rituximab has a potential therapeutic effect in this subgroup of patients without optimal responses to UDCA [5]. Another case reported on a 50-year-old patient with RA and PBC who was treated with rituximab and showed clinical and laboratory improvement in the first six months of treatment [12]. In our patient, we used a small amount of MTX (7.5mg/week) combined with two doses of 1000mg of rituximab, two weeks apart. After five months of follow-up, we observed a good clinical and biological response of her RA although there were persistent abnormalities in her liver tests.

The proinflammatory cytokine tumor necrosis factor alpha seems to play a major role in the pathogenesis of both RA and PBC. Spadaro *et al*. described the case of a 46-year-old patient with RA and PBC who was treated with anti-tumor necrosis factors [13]. During infliximab treatment, the authors observed a poor clinical response and persistence of liver function test abnormalities. After infliximab interruption, their patient’s levels of alkaline phosphatase dropped and had nearly reached normal values when etanercept was started. The etanercept maintained the patient’s liver enzyme levels within the normal range and controlled the arthritis during follow-up of 30 months, in contrast to the infliximab [13]. Another two cases have been reported where the patients were treated with etanercept and MTX 4mg/week, obtaining a significant improvement in disease activity and liver function, maintained one year after starting etanercept [14,15].

Our case report suggests that additional studies are needed to determine which treatment is safest and will demonstrate greater efficiency in both diseases.

## Conclusion

The association of RA and PBC is rarely reported in the literature. Studies are needed to determine the prevalence of this association. Recognition of the pathogenetic mechanism could have a tremendous impact on the appropriate care of patients and the choice of medication to treat both RA and PBC.

## Consent

Written informed consent was obtained from the patient’s next of kin for publication of this case report and accompanying images. A copy of the written consent is available for review by the Editor-in-Chief of this journal.

## Abbreviations

MTX: methotrexate; PBC: primary biliary cirrhosis; RA: rheumatoid arthritis; UDCA: ursodeoxycholic acid.

## Competing interests

The authors declare that they have no competing interests.

## Authors’ contributions

FL, FEA and TH analyzed and interpreted the patient data regarding the rheumatic and primary biliary cirrhosis disease. KB, NK and SM, contributed to writing the manuscript. All authors read and approved the final manuscript.
